# Supplementary material for: Improving access to specialist care in correctional facilities through Ontario eConsult
Source: PLoS One. 2024 Dec 3;19(12):e0314771. doi: 10.1371/journal.pone.0314771 (PMC11614223; doi:10.1371/journal.pone.0314771)
Supplement: Attachment — Submitted filename: Response to Reviewers.docx [file pone.0314771.s001.docx]

**Response to Reviewers**

PONE-D-24-21042

Improving access to specialist care in correctional facilities through Ontario eConsult

PLOS ONE

| **Comment** | **Response** |
| --- | --- |
| Editor Comment 1  Of particular note, one of the reviewers highlights the importance of using humanizing language for people/patients who are incarcerated. The US Centers for Disease Control and Prevention offer a concise list of recommended language: <https://www.cdc.gov/healthcommunication/‌Preferred_Terms.html> | We have addressed the use of the term 'offender' throughout the manuscript, replacing it with ‘people who are incarcerated’ or 'incarcerated individuals' to align with the recommended non-stigmatizing language for this this population. |
| Editor Comment 2  Please include the following items when submitting your revised manuscript:   - A rebuttal letter that responds to each point raised by the academic editor and reviewer(s). You should upload this letter as a separate file labeled 'Response to Reviewers'. - A marked-up copy of your manuscript that highlights changes made to the original version. You should upload this as a separate file labeled 'Revised Manuscript with Track Changes'. - An unmarked version of your revised paper without tracked changes. You should upload this as a separate file labeled 'Manuscript'. | - A rebuttal letter was uploaded and labeled “Response to Reviewers” - A marked-up copy of the manuscript was uploaded and labeled “Revised Manuscript with Track Changes” - An unmarked version of the revised manuscript without tracked changes was uploaded and labeled “Manuscript” |
| Editor Comment 3  Please ensure that your manuscript meets PLOS ONE's style requirements, including those for file naming. | The manuscript was revised to meet PLOS ONE’s style requirements. |
| Editor Comment 4  Please provide additional details regarding participant consent. In the ethics statement in the Methods and online submission information, please ensure that you have specified (1) whether consent was informed and (2) what type you obtained (for instance, written or verbal, and if verbal, how it was documented and witnessed). If your study included minors, state whether you obtained consent from parents or guardians. If the need for consent was waived by the ethics committee, please include this information. If you are reporting a retrospective study of medical records or archived samples, please ensure that you have discussed whether all data were fully anonymized before you accessed them and/or whether the IRB or ethics committee waived the requirement for informed consent. If patients provided informed written consent to have data from their medical records used in research, please include this information.  Once you have amended this/these statement(s) in the Methods section of the manuscript, please add the same text to the “Ethics Statement” field of the submission form (via “Edit Submission”). | A statement on the specified consent was added to the methods section. The same text was added to the Ethics Statement field of the submission form. |
| Editor Comment 5  In the financial disclosure, please state what role the funders took in the study. If the funders had no role, please state: "The funders had no role in study design, data collection and analysis, decision to publish, or preparation of the manuscript." Please include this amended Role of Funder statement in your cover letter; we will change the online submission form on your behalf.  If you would like to make changes to your financial disclosure, please include your updated statement in your cover letter. Guidelines for resubmitting your figure files are available below the reviewer comments at the end of this letter. | The role of the funders was added to the Financial Disclosure, and the amended Role of Funder statement was added to our cover letter. |
| Editor Comment 6  We note that you have provided funding information that is currently declared in your Funding Statement. However, funding information should not appear in the Acknowledgments section or other areas of your manuscript. We will only publish funding information present in the Funding Statement section of the online submission form. Please include your amended statements within your cover letter; we will change the online submission form on your behalf. | Funding information was removed from the Acknowledgements section and was added to our cover letter. |
| Editor Comment 7  In the Competing Interests section, please confirm that this does not alter your adherence to all PLOS ONE policies on sharing data and materials, by including the following statement: "This does not alter our adherence to PLOS ONE policies on sharing data and materials.” Please include your updated Competing Interests statement in your cover letter; we will change the online submission form on your behalf. | The statement was added to the Competing Interests section and is updated in our cover letter. |
| Editor Comment 8  Please confirm at this time whether or not your submission contains all raw data required to replicate the results of your study. Authors must share the “minimal data set” for their submission. PLOS defines the minimal data set to consist of the data required to replicate all study findings reported in the article, as well as related metadata and methods (https://journals.plos.org/plosone/s/‌data-availability#loc-minimal-data-set-definition).  For example, authors should submit the following data:  - The values behind the means, standard deviations and other measures reported;  - The values used to build graphs;  - The points extracted from images for analysis.  Authors do not need to submit their entire data set if only a portion of the data was used in the reported study.  If your submission does not contain these data, please either upload them as Supporting Information files or deposit them to a stable, public repository and provide us with the relevant URLs, DOIs, or accession numbers. For a list of recommended repositories, please see https://journals.plos.org/plosone/s/recommended-repositories.  If there are ethical or legal restrictions on sharing a de-identified data set, please explain them in detail (e.g., data contain potentially sensitive information, data are owned by a third-party organization, etc.) and who has imposed them (e.g., an ethics committee). Please also provide contact information for a data access committee, ethics committee, or other institutional body to which data requests may be sent. If data are owned by a third party, please indicate how others may request data access. | A minimal dataset cannot be provided due to the potential presence of identifiable information and because the data is owned by a third-party organization (Ontario Health).  The data used in this study is provided by Ontario Health (OTN Business Unit) (OH (OTN)) through a Data Sharing Agreement with the Ontario eConsult Centre of Excellence (COE), the lead organization for the Ontario eConsult program. OH (OTN) supplies the COE with transactional and record-level data generated through the use of the Ontario eConsult service. This data is used to generate reports on the utilization of the program, facilitating troubleshooting and supporting the ongoing development of the eConsult program. The authors have obtained permission to use the data set through a formal Data Sharing Agreement between Ontario Health (OTN Business Unit) and the Ontario eConsult Centre of Excellence, the lead organization for the Ontario eConsult program. This agreement ensures compliance with privacy regulations and outlines the terms of data access and usage. The authors did not receive any special privileges in accessing the data beyond the terms outlined in the Data Sharing Agreement. All data access and usage were conducted in accordance with the agreement, ensuring that the same terms apply to other researchers seeking access to the data.  To apply for access to the data, individuals can contact the Ontario Health (OTN Business Unit) Privacy Office through the following methods:  • Phone: 1-855-654-0888  • Email: [privacy@otn.ca](mailto:privacy@otn.ca)  Requests for de-identified aggregate Ontario eConsult utilization data can also be made through the following form: <https://form.asana.com/‌?k=zmEBJuRNGb9_YLGOXyqYLg&d=‌423266023889752> |
| Reviewer #1 Comment 1  I was curious about the sentence acknowledging poorer health outcomes among offenders and the statement about latent TB, sexually transmitted infections, Hep C, HIV and other blood borne infections, does this sentence capture the poorer health outcomes experienced by offenders? I would have expected poorer physical health, mental health and premature mortality more broadly among the group. | We have revised the literature in the introduction to better capture the poorer health outcomes experienced by incarcerated individuals. This includes citations that compare their health outcomes—such as physical health, mental health, and premature mortality—with those of the general population. We appreciate your input in helping us provide a more comprehensive understanding of this important issue. |
| Reviewer #1 Comment 2  Can you provide a couple of examples of the single speciality eConsult services operating in correctional facilities. | We have included examples of single specialty eConsult services currently operating in correctional facilities in the introduction of the revised manuscript. |
| Reviewer #1 Comment 3  It is not clear what the average number of eConsults sent per 1,000 offenders adds to the paper, especially considering the associated limitation outlined in the limitations section. Further clarification and explanation are needed in the methods and results sections about eConsults sent per 1,000 offenders, this would help clarify the statement in the discussion on page 13 that the “The volume of eConsults increased by 59% from the fiscal year 2019-2020 (pre-COVID-19) to 2020-2021…”. | Upon further reflection, we agree that the comparison does not contribute meaningfully to the paper, and we have decided to remove it. Our study focuses solely on volume of eConsults in Ontario’s correctional facilities rather than a comparison with the general public. Therefore, presenting cases per 1,000 is not adding to the paper, especially given the associated limitations. |
| Reviewer #1 Comment 4  I wondered if a table comparing the top 5 or so specialties for this setting with those at the provincial level would be of interest to readers. | The top five specialties accessed by the general public are dermatology (15%), hematology (8%), obstetrics and gynecology (7%), endocrinology and metabolism (7%), and internal medicine (6%). We highlighted the only key differences from the general population in the discussion. Specifically, cardiology was the top specialty accessed in the correctional setting, accounting for half of all eConsults, while dermatology, the most frequently accessed specialty by the general public, accounted for 15%. And that cardiology ranks 8^th^ among the general public. |
| Reviewer #1 Comment 5  On page 7. Two instances where “renumeration” should be “remuneration”. | We have corrected both instances of 'renumeration' to 'remuneration' on page 7. |
| Reviewer #2 Comment 1  Although I appreciate that in the world of prisons, "offender" is the term used to describe people, this term has not been common in research literature for at least a decade. I found the overall approach to this paper as set up in the introduction to be related to cost-savings rather than actually giving people who are incarcerated what they deserve. | We have addressed the use of the term 'offender' throughout the manuscript, replacing it with ‘people who are incarcerated’ or 'incarcerated individuals' to align with the recommended non-stigmatizing language for this this population. Additionally, we have highlighted the importance of access to care and its impact on improving outcomes for people who are incarcerated in the introduction. |
| Reviewer #2 Comment 2  The actual research aspect of this paper is limited: it is a descriptive study showing before/after uptake of speciality consults. There is no discussion of the systems used to educate clinicians about the EConsult, or evaluations of how the use of econsult different between clinicians or institutions. | The primary objective of this paper was to examine eConsult use within this specific setting (correctional facilities) and population (incarcerated individuals). However, we have published over 100 peer reviewed papers addressing the value of the eConsult service including educational value, its utilization, and its effects on access to specialist care across several Canadian jurisdictions. Additionally, we have also thoroughly explored the types of clinical questions posed through eConsult across various specialties. For a comprehensive list of our publications, please refer to our [website](https://www.champlainbaseeconsult.com/publications): [https://www.champlainbaseeconsult.com/‌publications](https://www.champlainbaseeconsult.com/publications) |
| Reviewer #2 Comment 3  There is no discussion about the problems with eConsult for people in jail/prison or their opinions of it. My experience as a clinician specialist going to the jails/prisons is that people who are incarcerated prefer to see someone in person. They feel they get better care. It is more humanizing. I could find NO voice from the perspectives of any one incarcerated as a threat in this whole paper. | Thank you for your insightful comment. This is a great point and direction for future research. The acceptability of eConsults is indeed an important area to explore and would require different methodologies, such as surveys or interviews, followed by qualitative analysis. Understanding the perspectives of incarcerated individuals is crucial for providing patient-centered care and assessing patient experience. While this has not been done in the incarcerated population yet, a relevant study has been conducted by our team with the general population ([https://www.champlainbaseeconsult.com/‌_files/ugd/ac5147_750c12a1d8184014a78‌f11b059f0d24f.pdf?index=true](https://www.champlainbaseeconsult.com/_files/ugd/ac5147_750c12a1d8184014a78f11b059f0d24f.pdf?index=true)). Our current paper focuses on eConsult service utilization for people who are incarcerated, and as such focuses on the metrics of service utilization. |
| Reviewer #2 Comment 4  The first paragraph of the discussion iterates what was already reported in the introductions. It also takes up over a page and is incredibly dense. | We acknowledge that the first paragraph of the discussion was dense, and we have revised it to enhance clarity and readability. Additionally, we have removed any content that overlapped with the introduction. |
| Reviewer #2 Comment 5  The item "referral avoided" is in the Figure 4. What is "referral avoided"? | “Referral avoided” refers to instances where a primary care provider initially considered making a referral but ultimately decided against it due to the guidance provided by the eConsult. To clarify the survey responses, we have revised Fig 4 and expanded on each response in the figure for better understanding. |
| Reviewer #2 Comment 6  I cannot tell if the authors mean "eConsult" as a proprietary item? Does this mean sending a consult request electronically? Telehealth is going on all over the world for people who are in prison. Just one site I found describing this for 2021. <https://csgjusticecenter.org/2021/04/12/‌three-things-to-know-about-implementing‌-telehealth-in-correctional-facilities/>. It feels like the authors of the paper really did not investigate what is going on for telehealth in other setting before writing this paper. | Thank you for your inquiry. Some electronic consultation (eConsult) systems across the world are privatized/commercialized, however the Ontario eConsult Service described in this paper is not; it is a provincially available service offered through the Ontario Ministry of Health. It allows primary care providers (PCPs) to submit consult requests electronically to specialists across the province of Ontario. To clarify, eConsult is a type of telehealth that facilitates electronic asynchronous communication between clinicians over a secure, web-based platform. It enables PCPs to communicate and collaborate with specialists to get timely advice on how to manage their patients, often eliminating the need for an in-person visit with the specialist. We have added more detail in the introduction and methods section to better explain the eConsult process and its benefits. |
